# Spatial transcriptomics of fetal membrane—Decidual interface reveals unique contributions by cell types in term and preterm births

**DOI:** 10.1371/journal.pone.0309063

**Published:** 2024-08-19

**Authors:** Lauren S. Richardson, Mary Elise Severino, Rahul Chauhan, Weibin Zhang, Marian Kacerovsky, Suresh K. Bhavnani, Ramkumar Menon

**Affiliations:** 1 Division of Basic Science and Translational Research, Department of Obstetrics & Gynecology, The University of Texas Medical Branch at Galveston, Galveston, Texas, United States of America; 2 Department of Physiology, College of Medicine, University of the Philippines Manila, Manila, Philippines; 3 Sealy School of Medicine, The University of Texas Medical Branch at Galveston, Galveston, Texas, United States of America; 4 Department of Epidemiology and Biostatistics, School of Public Health, The University of Texas Medical Branch at Galveston, Galveston, Texas, United States of America; 5 Department of Obstetrics and Gynecology, University Hospital Hradec Kralove, Faculty of Medicine in Hradec Kralove, Charles University, Hradec Kralove, Czechia; BC Children’s Hospital, CANADA

## Abstract

During pregnancy, two fetomaternal interfaces, the placenta–decidua basalis and the fetal membrane–decidua parietals, allow for fetal growth and maturation and fetal–maternal crosstalk, and protect the fetus from infectious and inflammatory signaling that could lead to adverse pregnancy outcomes. While the placenta has been studied extensively, the fetal membranes have been understudied, even though they play critical roles in pregnancy maintenance and the initiation of term or preterm parturition. Fetal membrane dysfunction has been associated with spontaneous preterm birth (PTB, < 37 weeks gestation) and preterm prelabor rupture of the membranes (PPROM), which is a disease of the fetal membranes. However, it is unknown how the individual layers of the fetal membrane decidual interface (the amnion epithelium [AEC], the amnion mesenchyme [AMC], the chorion [CTC], and the decidua [DEC]) contribute to these pregnancy outcomes. In this study, we used a single-cell transcriptomics approach to unravel the transcriptomics network at spatial levels to discern the contributions of each layer of the fetal membranes and the adjoining maternal decidua during the following conditions: scheduled caesarian section (term not in labor [TNIL]; n = 4), vaginal term in labor (TIL; n = 3), preterm labor with and without rupture of membranes (PPROM; n = 3; and PTB; n = 3). The data included 18,815 genes from 13 patients (including TIL, PTB, PPROM, and TNIL) expressed across the four layers. After quality control, there were 11,921 genes and 44 samples. The data were processed by two pipelines: one by hierarchical clustering the combined cases and the other to evaluate heterogeneity within the cases. Our visual analytical approach revealed spatially recognized differentially expressed genes that aligned with four gene clusters. Cluster 1 genes were present predominantly in DECs and Cluster 3 centered around CTC genes in all labor phenotypes. Cluster 2 genes were predominantly found in AECs in PPROM and PTB, while Cluster 4 contained AMC and CTC genes identified in term labor cases. We identified the top 10 differentially expressed genes and their connected pathways (kinase activation, NF-κB, inflammation, cytoskeletal remodeling, and hormone regulation) per cluster in each tissue layer. An in-depth understanding of the involvement of each system and cell layer may help provide targeted and tailored interventions to reduce the risk of PTB.

## Introduction

The human fetal membranes—the amniochorion that lines the intrauterine cavity–play a significant role in the maintenance of pregnancy and the initiation of parturition at term and preterm [1–5]. The two-layered membrane comprises a single layer of amnion epithelium and a multilayered chorion trophoblast connected through a collagen-enriched extracellular matrix containing sparsely distributed mesenchymal cells [6]. Amniotic and chorionic mesenchymal cells in the extracellular matrix are in transit, undergoing epithelial–mesenchymal transition (EMT) and mesenchymal–epithelial transition (MET) through a recycling process to strengthen and rebuild the membranes [1, 7]. Multiple cellular-biological processes associated with fetal membrane cell layers can determine the pregnancy status and the timing of parturition [8–10]. Dysfunctional membranes have been associated with preterm birth (PTB, < 37 weeks gestation) and preterm prelabor rupture of the membranes (PPROM) [11, 12]; the latter is a disease of the fetal membranes [13].

The amnion and chorion layers develop independently at implantation and fuse to form a single unit by the second trimester of pregnancy to provide protection and structural integrity to the amniotic cavity [6,14]. The two cell layers have very unique structures and functions. Two well-reported cellular processes in the fetal membranes are senescence and a nonreversible state of EMT of amniotic cells [4]. The progressive deterioration of the membrane due to senescence and the nonreversible state of EMT can generate localized inflammation that propagates to the maternal tissues and triggers parturition [4, 15–17]. Other cell fate mechanisms like apoptosis, necrosis, pyroptosis, and autophagy have also been reported to be associated with the physiological and pathological states of the fetal membranes [18–23]. The contribution of cell fate–determining pathways is unique for each cell type and depends on the local “environment.” The amnion epithelium that is proximal to the fetus and the amniotic fluid, mesenchymal cells in the matrix, and chorion trophoblasts that line with maternal decidua respond differently and contribute distinctly to the functional status of the fetal membranes [24, 25].

Several reports have indicated the contributions of each cell type to membrane physiology or pathology [26, 27]. However, studies that use single cell types (from either the amnion or chorion) or animal models do not provide an accurate anatomical representation of the human amniochorion with decidua. Hence, the available research has not yielded the data required to understand the biomechanical and physiological events that can predispose membranes to a dysfunctional status, a physiological necessity that promotes normal term labor but a pathological cause for preterm labor and PPROM. Amniotic epithelial cells exist in a metastate, and they are prone to transition due to endogenous and exogenous changes in their environment, whereas chorionic trophoblasts are highly resistant [7]. The objective of our project is to accurately determine the contributions of each cell type in their space to elucidate their function in determining pregnancy outcomes. We postulate that functionally and anatomically distinct amnion and chorion layers that form the feto-maternal interface with decidua participate differently regarding the contribution of the membrane to normal term and preterm parturitions.

Comprehensive knowledge of spatially resolved information that can determine the mechanistic functions of fetal membranes is essential to better understanding the contributions of the amnion and chorion to various processes during pregnancy and parturition. The amniochorion is a heterogenous mix of cells and contains amniotic epithelial cells, the amnion, chorionic mesenchymal cells, and chorionic trophoblasts along with maternal decidua to form the amniochorion-decidual interface (feto-maternal interface) [1]. Spatial transcriptomics allows visualization of gene expression patterns in three dimensions within cells in their native state. Recent advances in spatial transcriptomics has allowed researchers to determine the number of gene transcripts at distinct spatial locations in amniochorion tissue taken from term births and PTBs. The expression profile of each gene within a cell indicates its biological properties. Using the GeoMx Digital Spatial Profiler (Nanostring) with variable spatial resolution at the single-cell level, we obtained unbiased, spatially resolved transcriptomes of the feto-maternal interface from various pregnancy-associated conditions. We subjected fetal membranes with decidua from term not in labor (TNIL), term in labor (TIL), spontaneous PTB, and PPROM to spatial transcriptomics analysis. We analyzed the data by using unsupervised bipartite network analysis to visualize and decipher the functional contributions of various cell types in each condition.

## Methods

### Fetal membrane sample collection

Fetal membrane samples from four groups were collected from John Sealy Hospital, the University of Texas Medical Branch (UTMB) (consent is not required in line with UTMB local Ethics committee guideline for using human tissue that are, deidentified, discarded medical specimens), Galveston, Texas, and University Hospital Hradec Kralove, Czech Republic (All women in this study provided written informed consent prior to the collection of the fetal membranes and placental samples. Collection of the samples for the research was approved by the Institutional Review Board of the University Hospital Hradec Kralove (samples collected between November 2018 and November 2021) (April 2017, No. 201706 S17P). All women were Caucasian): (1) TNIL, term pregnancies (37–40 weeks of gestation) with no labor, who underwent elective caesarian section; (2) TIL, term pregnancies, with spontaneous labor, subsequent spontaneous membrane rupture, and normal vaginal delivery; (3) PTB, preterm pregnancies (28–36 weeks of gestation) with spontaneous labor, subsequent spontaneous membrane rupture, and normal vaginal delivery; and (4) PPROM, preterm pregnancies with the absence of labor before spontaneous rupture of the membranes. Membranes from women with pregnancy complications such as multiple gestations, placenta previa, preeclampsia, eclampsia, and fetal anomalies were excluded. Infection was not used as an exclusion criterion for preterm births during this study. The amniochorion was dissected from the midzone portion of the membranes (2 inches below the point of rupture and 2 inches above the placenta) and washed with normal saline. Blood clots and decidua were thoroughly removed using cotton gauze. Three 6mm biopsies per sample were placed in 10% neutral-buffered formalin for further tissue processing.

### Tissue processing and microarray preparation

**Fig 1:**
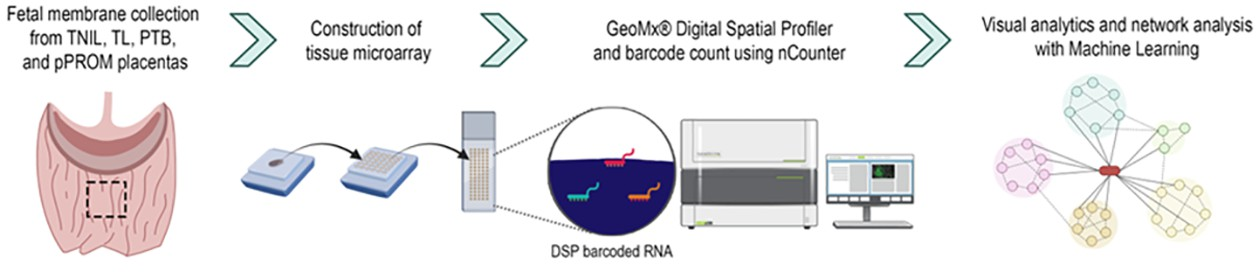
Tissue collection and spatial transcriptomic analysis experimental flow. Graphic schematic depicting the tissue collection step, microarray assembly, data collection by the spatial profiler, and downstream analysis.

After 24 h in 10% neutral-buffered formalin, tissues were transferred to 70% ethanol and then dehydrated and paraffinized by several changes of ethanol, xylene, and paraffin. Paraffin-embedded tissues were cut into 5-μm sections and stained with hematoxylin and eosin (H&E). Circular areas of the fetal membrane (2 mm in diameter) were selected using the corresponding H&E slide for each block and transferred to a tissue microarray block (Fig 1). The UTMB Anatomic Pathology Laboratory prepared the tissue microarray. The tissue microarray paraffin block was cut into 5-μm sections and immediately sent for slide processing for the GeoMx^®^ Digital Spatial Profiler (Fig 1).

### Digital spatial profiling and analysis

The GeoMx^®^ Digital Spatial Profiler is a novel platform developed by Nanostring that allows multiplex spatial profiling of RNA suitable for use on formalin-fixed, paraffin-embedded samples. In brief, the protocol consisted of slide preparation of tissue microarray of fetal membranes, antigen retrieval, staining with immunofluorescent markers (PanCK as an epithelial marker, CD45 as an immune cell marker, and SYTO13 for DNA staining), hybridization of tissue with ultraviolet (UV)-photocleavable oligonucleotide probes, scanning, region of interest (ROI) selection, probe collection, library preparation, and sequencing. Tissues were hybridized with the GeoMx^®^ Human Whole Transcriptome Atlas (Nanostring) panel of probes corresponding to over 18,000 protein-coding human genes based on the Human Gene Nomenclature Committee (HUGO) database cross-referenced with available messenger RNA (mRNA) sequences in the National Center for Biotechnology’s Information (NCBI) RefSeq database. Following hybridization, the slides were scanned on the GeoMx instruments with the workspace for ROI selection. ROIs of the amnion epithelial layer (AEC), amnion mesenchymal layer (AMC), chorion trophoblast layer (CTC), and decidua (DEC) were selected individually for each tissue sample, resulting in 48 total ROIs. The collection was initiated with UV-light exposure of the ROIs to cleave the UV-sensitive probes. The released probe-specific Digital Spatial Profiler barcodes were aspirated and collected into the 96-well Digital Spatial Profiler plates. Indexing oligos were hybridized to Nanostring optical barcodes for digital counting on the nCounter. BioCenter performed slide processing and library preparation and sequencing at University of Texas Southwestern Medical District.

### GeoMx NGS library preparation and sequencing

The NGS libraries were prepared per the manufacturer’s guidelines. In brief, after the collection was complete, aspirates in the collection plate were dried at 65°C for 1 hour in a thermal cycler with the lid open and resuspended in 10 μL of nuclease-free water. 4 μL of rehydrated aspirates were mixed with 2 μL of 5×PCR Master Mix and 4 μL of SeqCode primers, and PCR amplification with 18 cycles was performed. The PCR products were then pooled equally and purified twice with 1.2×AMPure XP beads (Beckman Coulter). The final libraries were evaluated and quantified using Agilent’s high sensitivity DNA kit and Invitrogen’s Qubit dsDNA HS assay, respectively. Total sequencing reads per DSP collection plate were calculated using the NanoString DSP worksheet. The libraries were subjected to 2×38 bp paired-end sequencing on an Illumina NovaSeq 6000 system with a 100-cycle S1 kit (v1.5).

### Data collection

The data included 18,815 genes from 13 patients—TIL (n = 3), PTB (n = 3), PPROM (n = 3), and TNIL (n = 4)—expressed across four layers (AEC, AMC, CTC, and DEC). After quality control by NanoString, there were 11,921 genes and 44 samples. The data were processed in two ways: (1) by Nanostring to evaluate hierarchical clustering of cases and controls combined, and (2) by our group to evaluate heterogeneity within the cases. Two forms of analysis were done to evaluate the data with and without the inclusion of controls in the same cluster analysis, as this can strongly impact the data outcomes.

### Nanostring data analysis [28, 29]

Hierarchical clustering was conducted on the top 10% most variable genes based on coefficient of variation (CV; see the formula below). Scaled log2-transformed normalized expression values were plotted and clustered using average linkage based on Pearson distances. Heatmaps of the top differentially enriched pathways for each comparison group were also generated. A heatmap plotted ssGSEA Gene Set Variation Analysis (GSVA), which is a Bioconductor/R tool [30] enrichment scores for each ROI. The columns were clustered using complete linkage based on Euclidean distances. The bars on the rows display enrichment intensity (i.e., fold change), and the colors correspond to the group exhibiting the enrichment.

### Data analysis focusing on heterogeneity

To analyze heterogeneity within the cases, we divided the samples into cases (TIL, PTB, and PPROM) and controls (TNIL) and used the following steps to analyze the data.

#### Feature selection

For each gene, we calculated its CV across the 44 samples using the following formula: CV = {SD [log2(X)]} / {Mean [log2(X)]}, where X is the normalized count of the gene after quality control and SD is the standard deviation. Next, we selected the top 10% (d = 1192) of the genes based on the CV.

#### Bipartite network analysis

We modeled the data as a weighted bipartite network [28] where nodes consisted of either cases (n = 33) or genes (d = 1192), with weighted edges connecting them. We calculated the edge weight as log2(X), where X is the normalized count of the respective sample-gene pair. Next, we used bipartite modularity maximization [29, 30], considering the edge weights, to identify the number of biclusters, their members, and the degree of biclusteredness using bicluster modularity (Q, intuitively defined as the fraction of edges falling within clusters, minus the expected fraction of such edges in a network of the same size with randomly assigned edges). Then, we measured the significance of Q by comparing it with a distribution of the same quantity generated from 1000 random permutations of the network while preserving the network size (the number of nodes) and the distribution of weighted edges for each gene.

#### Enrichment analysis

We conducted an enrichment analysis of the cases and genes in each bicluster using R version 4.3.0. For the cases, we plotted the number of cases from each layer. Furthermore, we used chi-square to measure the difference in the distribution of layers between each pair of biclusters and identified pairs that were significantly different (two-tailed test using p < 0.05) after correcting for multiple testing. For the genes, we used Ingenuity pathway analysis (IPA; Qiagen, Hilden, Germany) to identify protein networks that included the top 10% differently expressed genes from each of the four identified clusters.

#### Interpretation of preterm heterogeneity

We used the Fruchterman-Reingold [32] and Explode Layout [33, 34] algorithms to visualize the bipartite network, with cases shown as circles, genes shown as triangles, and the case–gene associations shown as weighted edges. Furthermore, we used arrows to display on the network which pairs of biclusters were significantly different based on the distribution of their layer profile. An expert panel of three biologists used the integrated plot to interpret the molecular heterogeneity in PTBs and to generate testable hypotheses for future clinical translational applications.

## Results

### Histological analysis of term and preterm fetal membrane feto-maternal interface tissue

**Fig 2:**
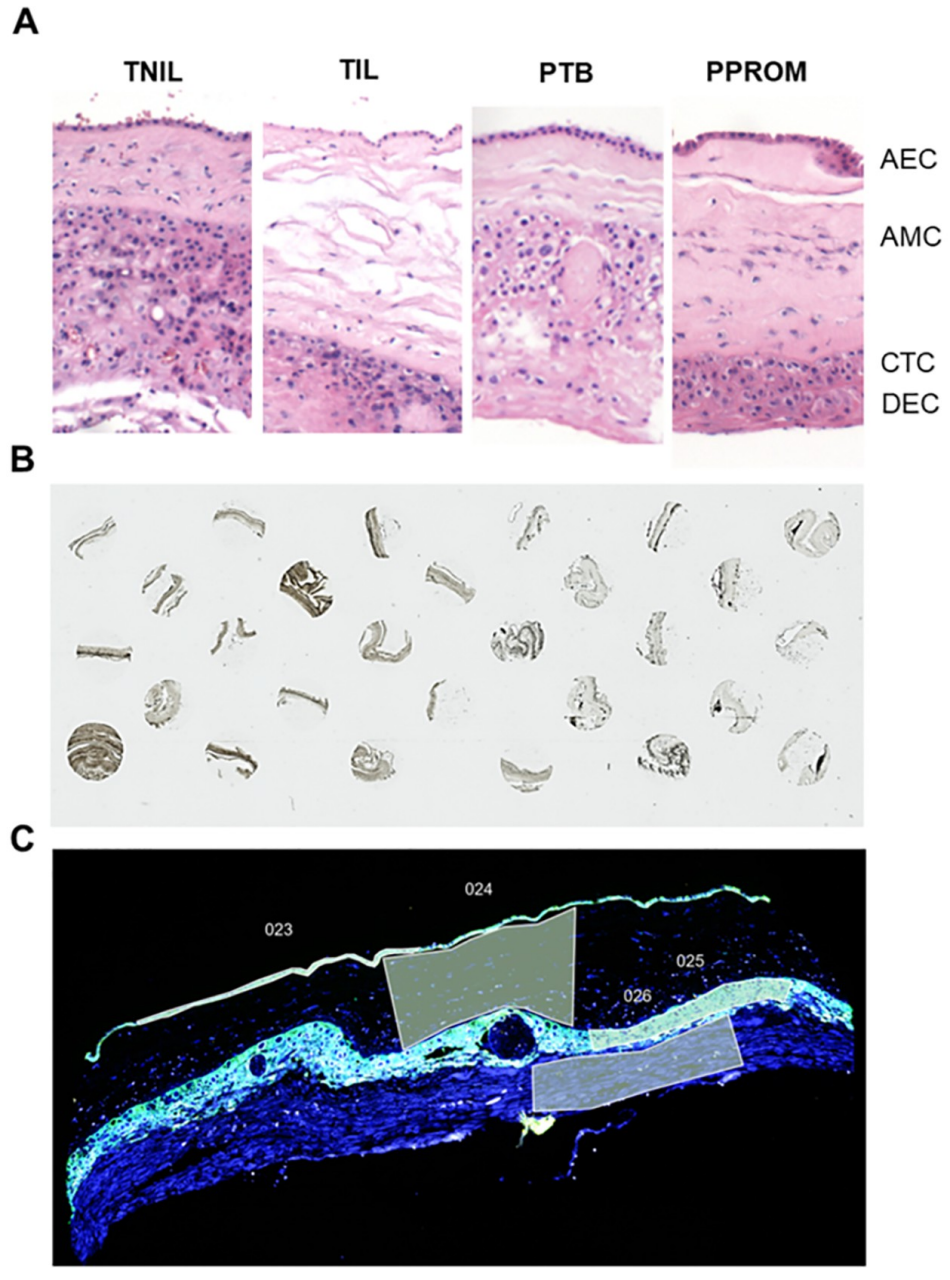
Histological analysis of the fetal membrane feto-maternal interface. **A)** H&E stain of the feto-maternal interface during different term or labor conditions highlighting the location of the amnion epithelial cells (AEC), amnion mesenchymal cells (AMC), chorion trophoblast cells (CTC), and maternal decidua (DEC). 10x image. **B)** Pan-cytokeratin statin (brown) histology microarray documenting the placement of the tissue samples. Brightfield microscopy, stritched image, 2x. **C)** Fluorescent image documenting pan-cytokeratin stain (green) and cell nuclei (DAPI; blue) within the tissue. Example, regions of interest of the AEC (023), AMC (024), CTC (025), and DEC (026), are shown as to how they would be selected for sequencing.

Histopathological analysis of fetal membranes used for spatial transcriptomics revealed no discernible differences in the overall structure of the amnion, chorion, and decidua among the studied groups (Fig 2). However, closer examination of the choriodecidua in the PPROM group revealed a notable increase in leukocytic infiltration compared with the other groups, suggesting an inflammatory component that is more apparent in PPROM. Furthermore, the PPROM membranes exhibited distinct histological characteristics in the chorion, notably an increased presence of vacuolated laeve cells, which were not as prominent in the other groups. Importantly, the sampled areas consistently demonstrated intact single-cell layer amnion epithelial membranes across all groups. While our gross observations did not reveal significant differences in histological features, it is plausible that disparities at the transcriptional level may underlie the observed clinical distinctions and warrant further investigation. For this study, these membranes did not undergo the detailed electron microscopic or multiphoton imaging analysis as it is previously reported [11, 24, 31, 32].

### Differential clustering of transcriptional regulation between term and preterm labor tissue

**Fig 3:**
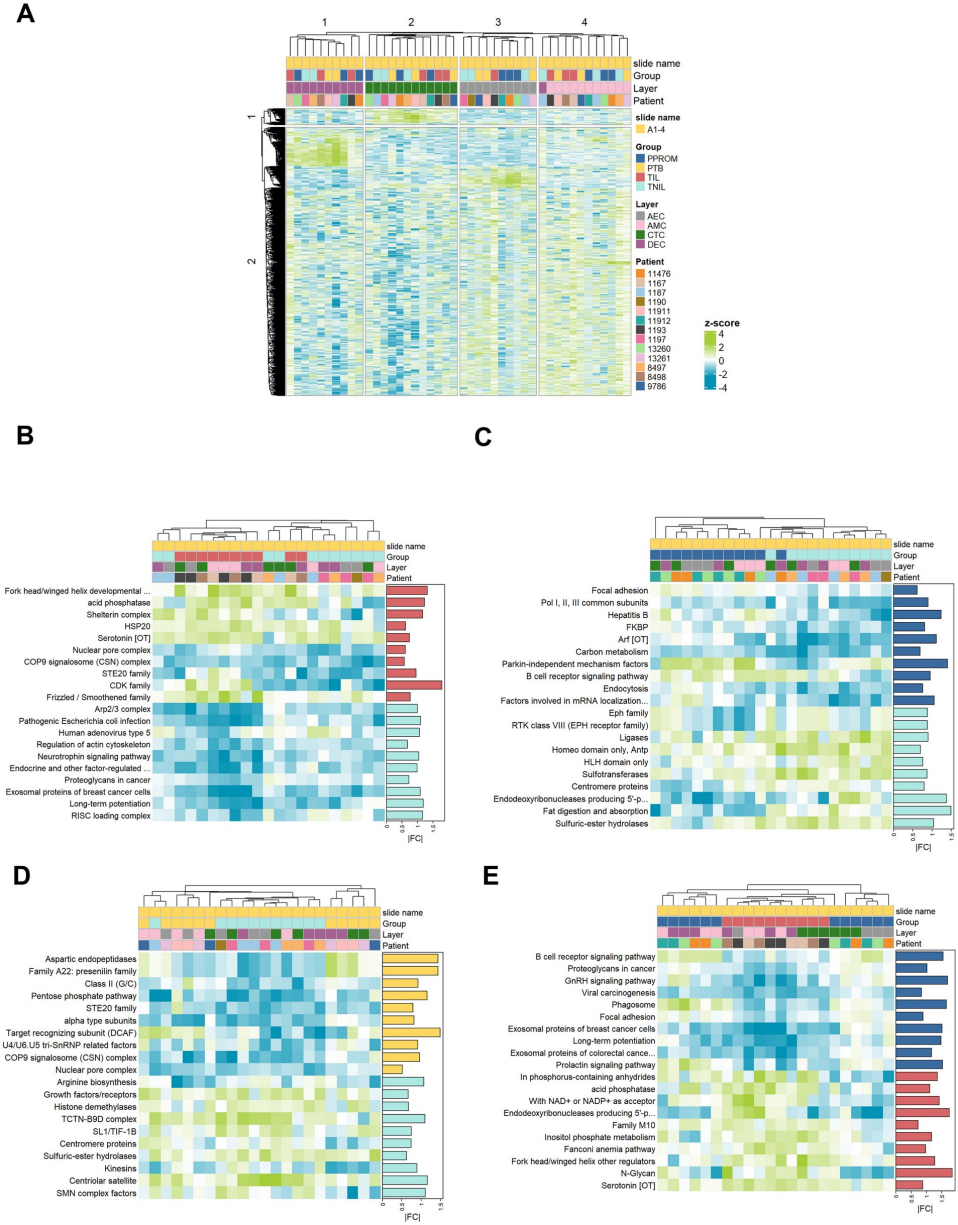
Hierarchical clustering of both cases and controls combined. **A)** Hierarchical clustering of the top 10% most variable genes based on coefficient of variation. Scaled log2 transformed normalized expression values are plotted and clustered using average linkage on Pearson distances. Heatmaps of the top differentially enriched pathways for each comparison group: TIL vs. TNIL **(B)**, PPROM vs. TNIL **(C)**, PTB vs. TNIL **(D)**, PPROM vs. TIL **(E).**

The unsupervised hierarchical clustering of ROIs based on Pearson distances of normalized data z-scores revealed distinct clustering patterns, reinforcing that each layer is transcriptionally unique from the others (Fig 3A). When we compared TNIL (light blue) and TIL (red) ROIs and TNIL and PPROM (dark blue) ROIs, we identified differential expression of labor-related pathways (Fig 3B; S1 Fig) (Fig 3C; S2 Fig). However, when we compared TNIL and PTB (yellow) ROIs, we did not observe strong contrasting differences in their transcriptional profiles (Fig 3D). In contrast, comparison of TIL and PPROM ROIs demonstrated differential expression of several signaling pathways (Fig 3E), highlighting that TIL and PPROM are indeed transcriptionally distinct, which has been documented by others in the field [33–36].

### The spatial resolution of feto-maternal interface layer contributions to term and preterm labor signaling

**Fig 4:**
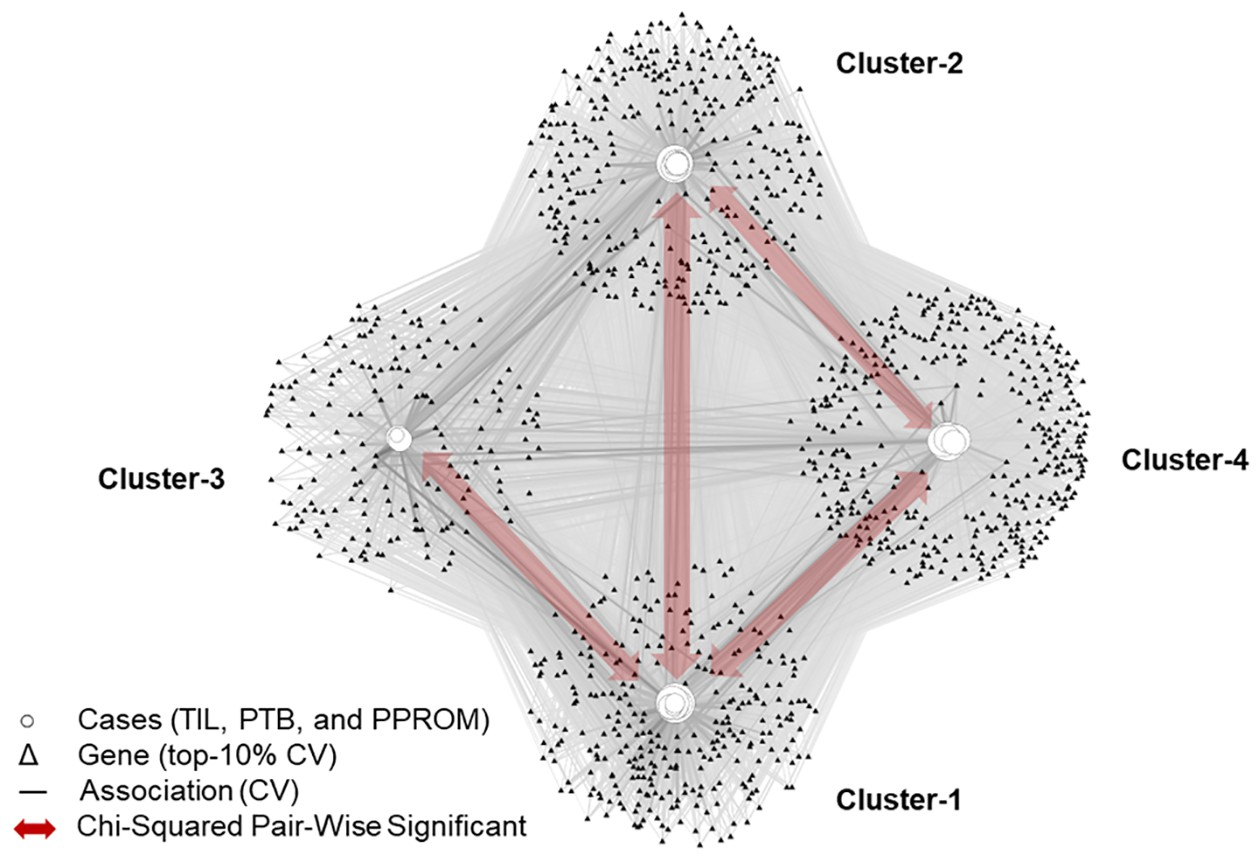
Identification of heterogeneity within cases (TIL, PTB, and PPROM). Results of the bipartite network analysis of cases (n = 33) and genes (d = 1192) identified four clusters with significant clusteredness, suggesting strong heterogeneity within the cases.

The bipartite network analysis of cases (n = 33) and genes (d = 1192) identified four biclusters (hereafter referred to as clusters), with significant biclusteredness (Q-Real = 0.047, Q-Random = 0.032, Z = 40, P < 0.001, two-tailed) as measured by modularity maximization (Fig 4). Table 1 shows the pairs of clusters that were significantly different from each other based on their layer profile of cases (yellow highlights in Table 1 and red arrows in Fig 4). These results suggest hypotheses for which inflammatory pathways are triggered within and across feto-maternal interface layers, as discussed blow.

**Table 1:** Pairs of clusters that were significantly different from each other based on the layer profile of their cases.

Cluster_i	Cluster_i	Chi_square	Degree_of_freedom	p_value	p_Bonferroni
1	2	19	3	0.00027	0.00164
1	3	11.8	2	0.00271	0.01626
1	4	14.5	2	0.00071	0.00426
2	3	7.79	2	0.02618	0.15706
2	4	14.2	2	0.00083	0.00498
3	4	2.34	1	0.1261	0.75663

**Fig 5:**
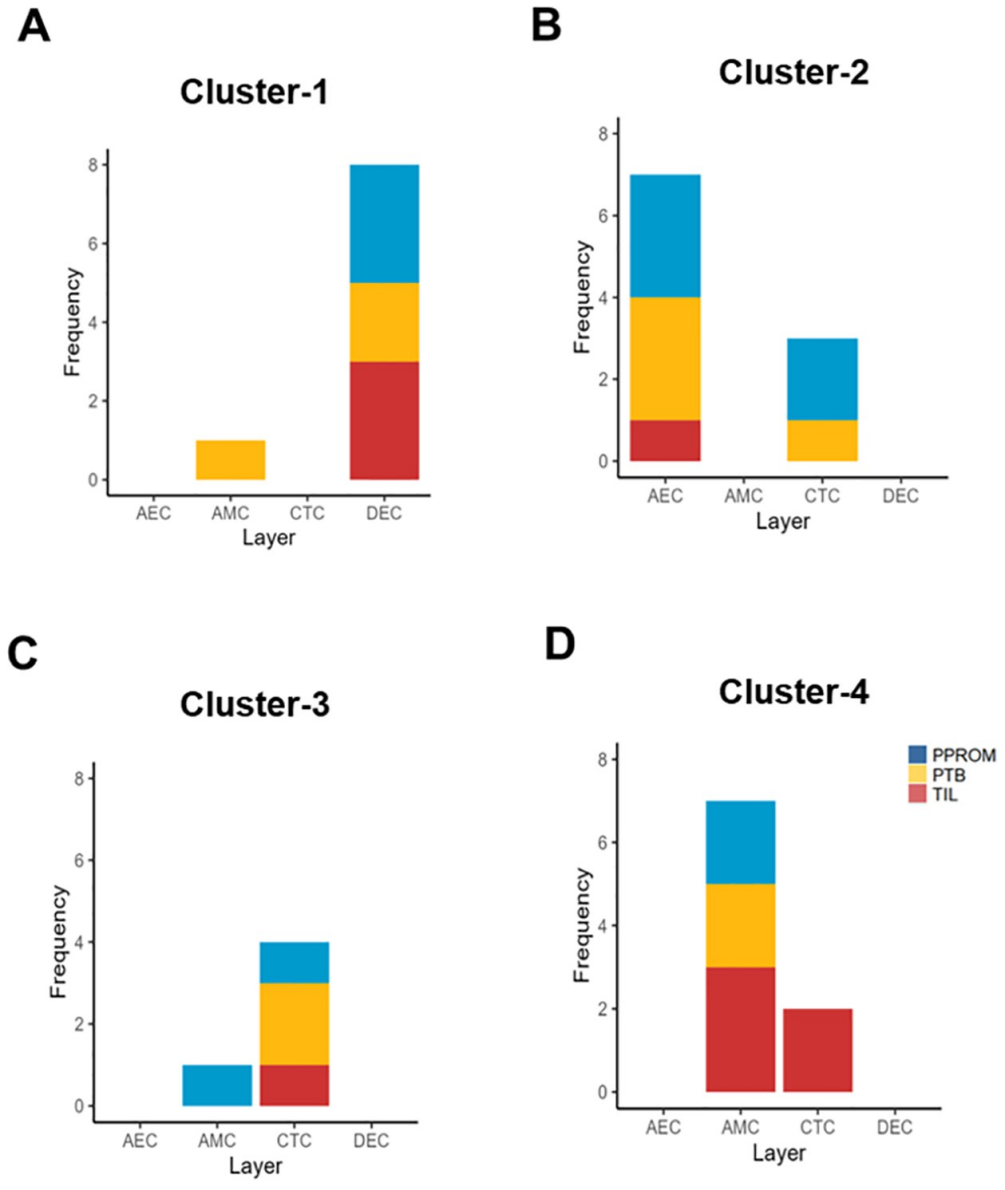
Breakdown of clusters into cell layer and case phenotype. **A-D)** Clusters are broken down into graphs first highlighting the frequency of genes from clusters-1-4 in each cell layer (AEC, AMC, CTC, DEC) and the frequency of genes in each case condition.

Genes within clusters correlated with cellular layers and labor phenotypes (i.e., cases), showing strong pairwise differences in the different profiles. Cluster 1 genes were present predominantly in DECs in all labor phenotypes (Fig 5A). Cluster 2 genes were predominantly found in AECs in PPROM (blue) and PTB (yellow) (Fig 5B), while Cluster 3 centered around CTC genes in all labor phenotypes (Fig 5C). Interestingly, Cluster 4 contained AMC and CTC genes identified in term labor cases (Fig 5D).

**Fig 6:**
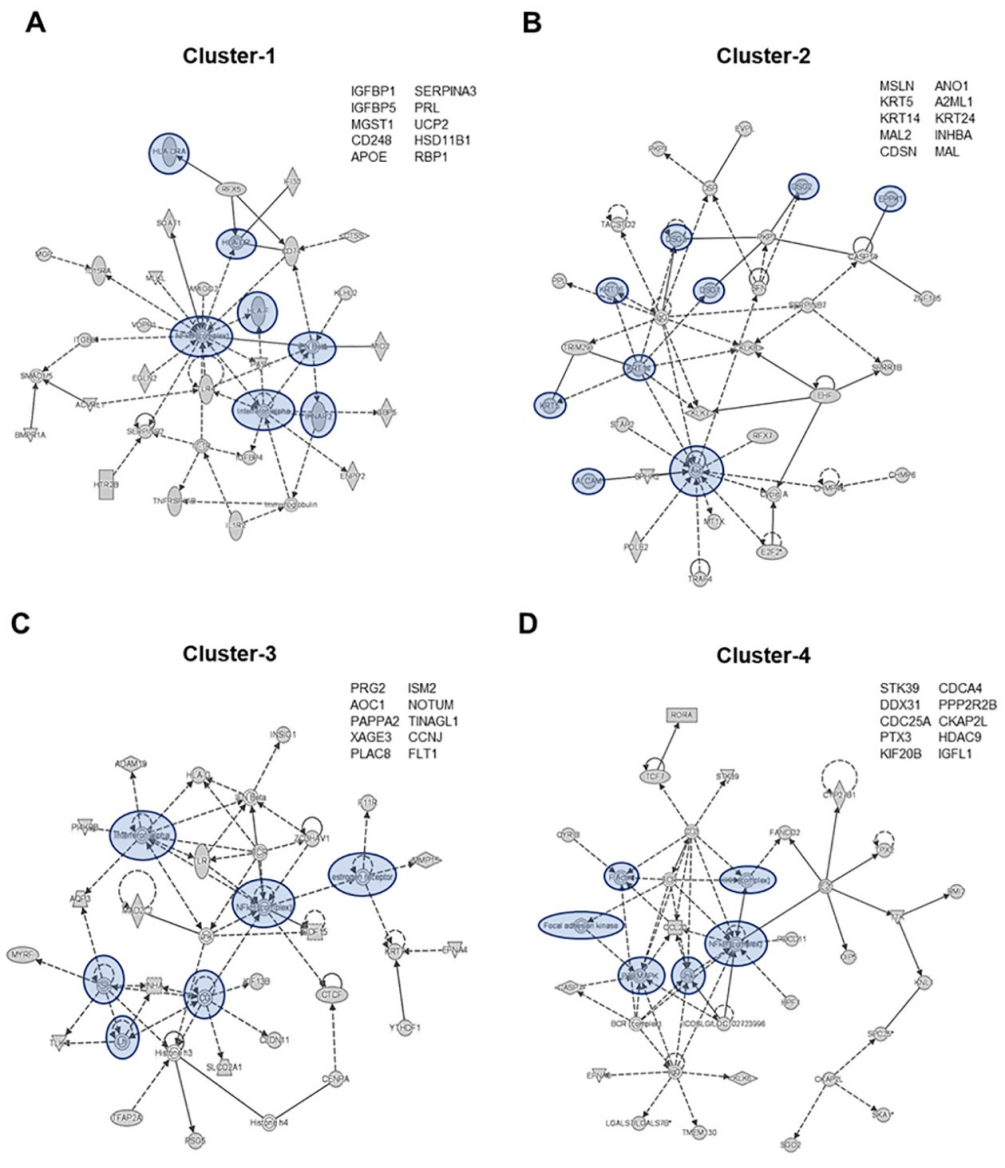
IPA analysis of genes within clusters to identify biologically relevant signaling pathways. **A-D)** The top signaling network for each cluster was identified in IPA and analyzed in its biological context based on the cell layer and term/labor phenotype identified within the cluster. The top-10 expressed genes, identified in the raw data, are shown in the upper right corner of each cluster’s network. Critical signaling molecules are highlighted in blue.

IPA of the top-10 genes revealed pathways mainly related to inflammatory processes (Fig 6). The top-10 highest expressed genes are shown next to each cluster’s case layer profile. Cluster 1 was dominated by cases from the DEC layer, and the corresponding genes formed a signaling network characterized by NF-κB-induced inflammation (Fig 6A). This inflammatory signaling was associated with interferon (i.e., interferon-α, interferon-β, and interferon receptors) and human leukocyte antigen (HLA; i.e., HLA-DRA, HLA-DR, HLA-F)-regulated pathways canonically associated with immune cell regulation. Cluster 2 was dominated by cases from the AEC and CTC layers, and the corresponding genes were associated with signaling pathways related to cell adhesion (i.e., activated leukocyte cell adhesion molecule [ALCAM], desmoglein 1 [DSG1], DSG2, DSG3, and epiplakin 1 [EPPK1]), and cytoskeletal reorganization (i.e., keratin 16 [KRT16], KRT14, KRT5) that regulate stress kinase (i.e., AKT) complexes (Fig 6B). Similarly to Cluster 1, Cluster 3 and Cluster 4 determined cell type–specific inflammatory signaling at the fetal membrane of the feto-maternal interface (Fig 6C and 6D). Cluster 3 was dominated by cases from the CTC layer, and the corresponding genes formed a signaling network that centered on interferon-α-induced NF-κB signaling, along with hormone regulation (i.e., luteinizing hormone [Lh], follicle-stimulating hormone [FSH], chorionic gonadotropin [CG], and estrogen) (Fig 5C). Cluster 4 was dominated by cases from the AMC layer and the corresponding genes centered around stress associated mitogen-activated kinase (i.e., p38 MAPK and JNK)-associated NF-κB signaling and cytoskeletal remodeling (i.e., F actin and focal adhesion kinases) (Fig 6D). Overall, IPA highlighted the differential inflammatory signaling pathways activated at the unique fetal membrane–maternal interface layers during term or preterm labor.

## Discussion

Spontaneous PTB or PTB following PPROM are complex syndromes mediated by a multitude of etiologic factors, pathophysiologic pathways, and manifestations of these pathways in different intrauterine organ systems [2, 37]. PTB and PPROM can be classified as a disease of the mother, a disease of the fetus, or both; however, the fetus is not often considered to be the patient because interventions are primarily directed toward maternal symptoms. Recently, we reviewed how each intrauterine organ can contribute to its own pathways, causing an adverse pregnancy outcome [38]. Therefore, PTB can be called a disease of the decidua, myometrium, or cervix on the maternal side, or may be referred to as the disease of the fetal membranes or the placenta on the fetal side [38]. We derived these theoretical concepts from the reports available based on a single system (mother or fetus) or organ analysis (placenta, decidua, etc.). In normal TIL, the signals of fetal readiness to have an independent life and the maternal response to such fetal signals to deliver the mature fetus are synchronized to promote labor [39–41]. Maternal risk factors pathologically override the physiological mediation at term [42]. The risk factors may have distinct impacts on different sites, and it is likely that not all intrauterine organs are involved initially in the pathological pathways that cause preterm labor. We postulate that PTB and PPROM are not merely an early activation of signals often seen at term. Regardless, the final pregnancy outcome depends on synchronized activity of each system (transitioning each organ from a quiescent state to an active state) that results in emptying of the uterus [38]. However, it is important to know the system- and organ-based involvement in the term and preterm labor processes and the mechanistic participation that can result in the final events. This knowledge of each system and organ involvement may help to provide targeted and tailored interventions to reduce risk. Single-cell transcriptomics data on the decidua [43, 44], myometrium [45], cervix [46], and placenta [47, 48] have yielded insights into the mechanisms. Fetal membranes are an understudied area of intrauterine research. In this study, we used a single-cell transcriptomics approach to unravel the transcriptomics network at spatial levels to discern the contributions of each layer of the fetal membranes and the adjoining maternal decidua.

Our visual analytical approach of spatially recognized differentially expressed genes allowed us to highlight the involvement of gene clusters, the top 10 differentially regulated genes, and their pathways in each layer of the fetal membranes and decidua and in each of the pregnancy conditions. Cluster 1 genes are predominantly represented by inflammation of decidual cells and, to a lesser extent, mesenchymal stromal cells of the membrane matrix. Cluster 2 genes are represented predominantly by the amnion epithelium and CTCs. Interestingly, it does not represent an inflammatory phenotype (i.e., pro-inflammatory cytokines, MMPs, prostaglandins) [49, 50] but rather cellular reorganization–associated signals. This is a classic response of amniochorionic cells under duress trying to maintain membrane homeostasis (i.e., cellular transitions) and structural integrity [7, 51–53]. Cluster 3 is represented by differentially regulated genes in the chorion trophoblast, which represent changes in the inflammatory and endocrine statuses. Cluster 3 also had minimal representation from the mesenchymal cells of the extracellular matrix. Cluster 4 was dominated by mesenchymal cells of the membrane matrix representing mitogen kinase pathway activation and associated NF-κB-mediated inflammation. Thus, in the fetal membrane–decidual interface, spatially separate regions of the same organ contribute distinctly to the overall inflammation, stress response, MAPK signaling, cellular reorganization, and endocrine involvement to facilitate the outcome. Of note, cluster 2 was defined by the amnion epithelium and chorion trophoblast transcriptomes, and it was primarily in membranes from PPROM (and to a lesser extent in membranes from PTB). This supports prior reports that have noted senescence, EMT, microfracture formation, and autophagy disturbance in these cell types and inflammation resulting from these biological mediations as the primary mediators of PPROM.

Our approach of using bipartite networks has quantitative and interpretive advantages. Quantitatively, unlike many clustering methods like k-means and hierarchical clustering, the method does not require user input to identify either the expected number of clusters before the analysis (as required in k-means) or to determine the expected number of clusters using *post hoc* methods (as done when using hierarchical clustering and dendrograms). Instead, the modularity maximization method uses an objective function to automatically identify the number of clusters and outputs the modularity (Q), which measures the strength of separation between the clusters. Furthermore, Q can be used to measure the significance of that separation compared with a null model using a permutation test. Interpretatively, the method employs a node-edge graph visualization of the associations and their clustering using force-directed layouts to rapidly examine complex patterns in the data. This is because the node-edge graph representation uses the proximity of multiple nodes from both sides of the data (cases and genes), enabling a more intuitive visualization of the data compared with heatmaps, which use a tabular representation that is more constrained. A node (row or column in the heatmap) can be immediately adjacent to a maximum of two other nodes on either side. However, the expressive power of bipartite networks requires sufficient rows and columns in the data to enable their effective use.

To be consistent with the heatmap analysis, we used the CV to identify which genes changed the most across all of the preterm and term conditions. In our analysis, this measure captured most of the genes that were the most differentially expressed compared with the controls. However, other measures, such as chi-square, could generate different results, and this issue should be examined in future research. Finally, we present the results in Table 1 mainly for hypothesis generation due to the small sample size.

Spatial transcriptomics and bipartite network analysis are not indicative of function, but they are indicative of a trend and useful to generate hypotheses for future work. Transcriptome data or even quantitation of specific transcripts alone are insufficient to draw conclusions on mechanisms, pathways, or biomarkers; therefore, we have not attempted to speculate more based on these data. Our data indicate the likely contributions of each layer in specific conditions, and it is confounded by labor- and rupture-associated factors. We have not stratified our analysis based on the type of risk (e.g., infectious inflammation or sterile inflammation) or any other socio-demographic factors because the sample size we used for this study is insufficient to perform any additional analyses. The availability of these data and the trends seen in the amniochorion–decidual interface may help to design future hypothesis-driven studies.

## Supporting information

S1 Fig Violin plots showing the differently expressed genes between TNIL and TL fetal membranes. (TIF)

S2 Fig Violin plots showing the differently expressed genes between TNIL and PPROM fetal membranes. (TIF)

S1 Checklist Human participants research checklist. (DOCX)
