# Supplementary figures and images for: Spatial transcriptomics of fetal membrane—Decidual interface reveals unique contributions by cell types in term and preterm births
Source: PLoS One. 2024 Aug 19;19(8):e0309063. doi: 10.1371/journal.pone.0309063 (PMC11332933; doi:10.1371/journal.pone.0309063)

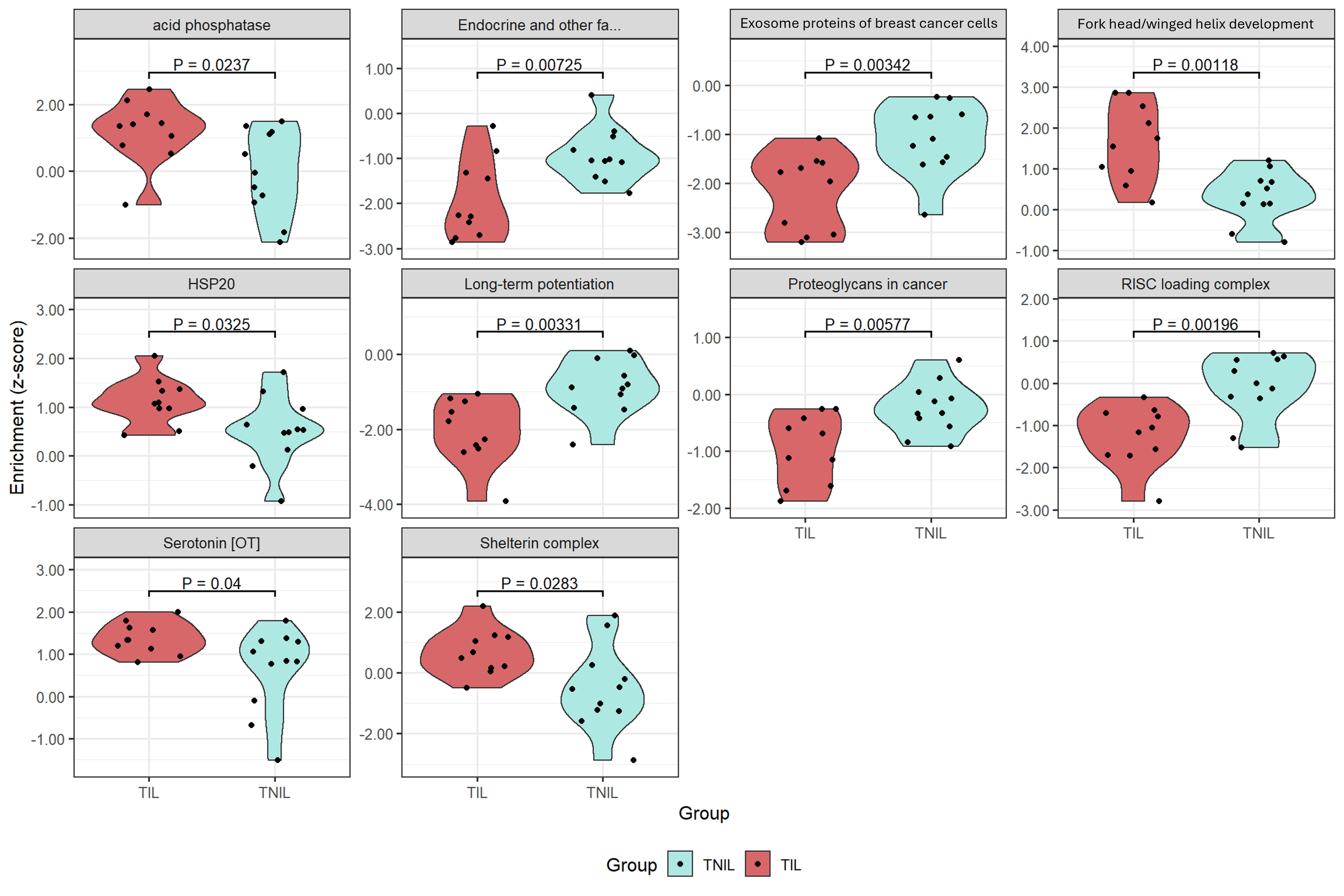

Supplement: S1 Fig — (TIF) [file pone.0309063.s001.tif]

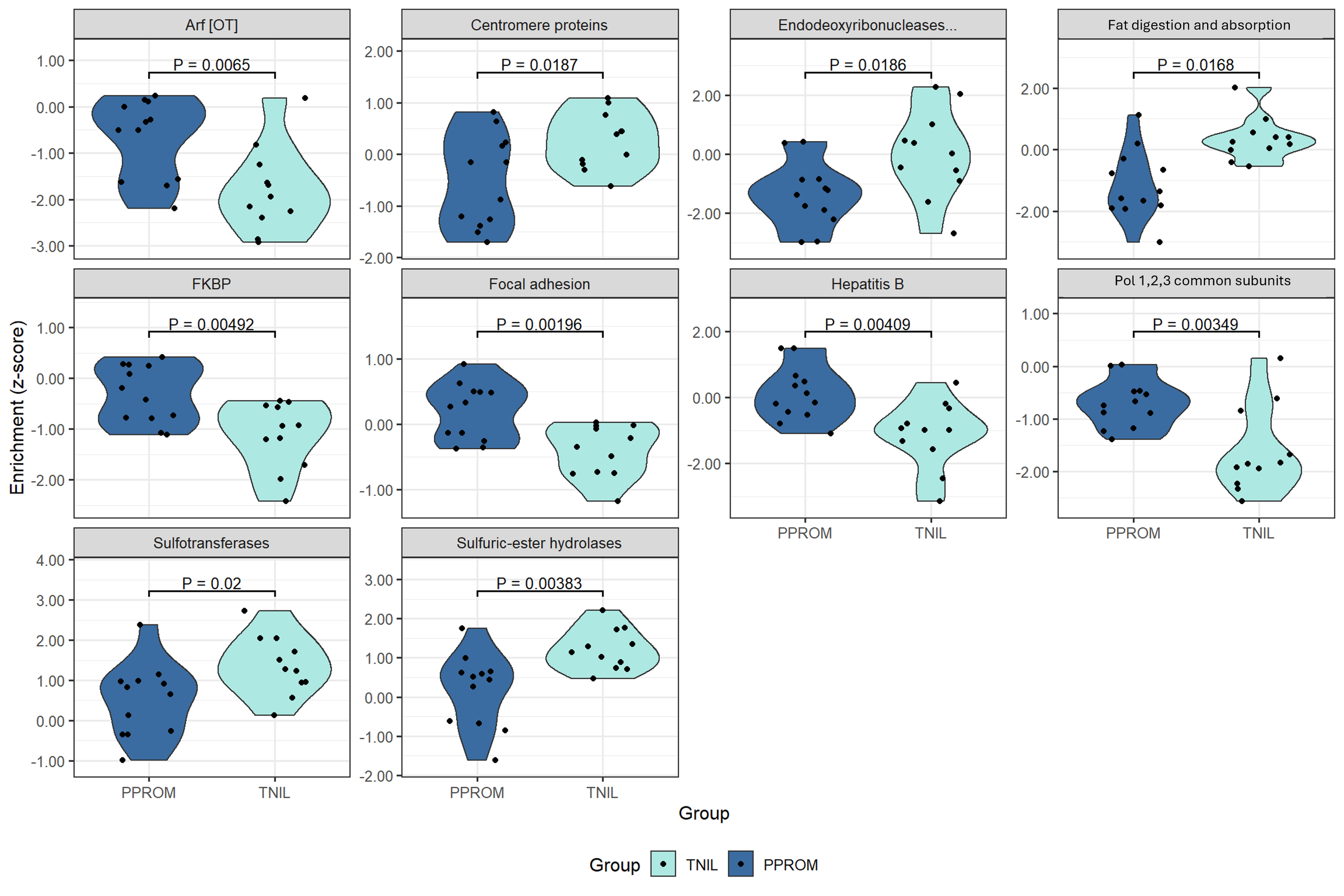

Supplement: S2 Fig — (TIF) [file pone.0309063.s002.tif]
